# Effects of a contoured foot orthosis and flat insole on plantar pressure and tibial acceleration while walking in defence boots

**DOI:** 10.1038/s41598-018-35830-5

**Published:** 2019-02-08

**Authors:** Daniel R. Bonanno, Ketharasarma Ledchumanasarma, Karl B. Landorf, Shannon E. Munteanu, George S. Murley, Hylton B. Menz

**Affiliations:** 1 Discipline of Podiatry, School of Allied Health, La Trobe University, Melbourne, Victoria, 3086 Australia; 2 La Trobe Sport and Exercise Medicine Research Centre, School of Allied Health, La Trobe University, Melbourne, Victoria, 3086 Australia

## Abstract

This study investigated the effects of a contoured, prefabricated foot orthosis and a flat insole on plantar pressure and tibial acceleration while walking in defence boots. Twenty-eight adults walked along an 8-metre walkway in a: (i) defence boot (control condition), (ii) defence boot with a flat insole, and (iii) defence boot with a contoured foot orthosis. Plantar pressure data were collected using the pedar-X in-shoe system and tibial accelerations were measured with an accelerometer. In relation to plantar pressure under the rearfoot, the contoured foot orthosis, compared to the defence boot, decreased peak pressure and maximum force, and increased contact area. Under the medial midfoot, the contoured foot orthosis and flat insole increased peak pressure, maximum force and contact area. Under the medial forefoot, the contoured foot orthosis and flat insole increased maximum force. Under the lateral forefoot, the contoured foot orthosis and flat insole increased contact area, with the flat insole also increasing maximum force. In relation to tibial acceleration, the contoured foot orthosis, compared to the defence boot, decreased tibial peak positive acceleration. These findings provide novel biomechanical evidence for the effects of contoured foot orthoses in defence boots.

## Introduction

Lower limb overuse injuries are common among physically active defence personnel^1–3^, with the incidence reported to range from 10 to 47% during initial defence training^2,4^. Common lower limb overuse injuries include medial tibial stress syndrome, patellofemoral pain, Achilles tendinopathy, plantar fasciitis, and stress fractures^1,5–8^. Numerous risk factors for lower limb overuse injuries during defence training have been identified, including older age, lower fitness levels, previous injury and some lower limb biomechanical variables^2,9–11^. Broadly, lower limb overuse injuries are proposed to develop in tissues that are exposed to loads that are greater than what the tissues are prepared for^12,13^. Accordingly, interventions that decrease tissue loads or increase the body’s tolerance to training loads are of interest to the defence forces.

Defence boots are primarily designed to protect the foot and ankle from direct impact, extreme environmental conditions, and acute musculoskeletal injuries^14^. However, defence boots have long been considered a potential contributing factor for overuse injury^15^. Defence boots are often heavy while also having a standard shape and design, which may lead to poor fit and discomfort^15^. In addition, laboratory-based studies have demonstrated that wearing defence boots can alter lower limb kinematics and also have deleterious effects on lower limb tissue loads and impact forces when compared to athletic footwear^14,16^.

Making substantial changes to defence boot design or using alternative footwear is not always feasible in the defence context. As such, shock-absorbing insoles or foot orthoses are often used in defence boots to improve comfort^17^, reduce impact forces^18^, and to alter lower limb function^14,16–18^. A recent systematic review concluded that prefabricated foot orthoses, but not shock-absorbing insoles, can decrease the incidence of overall lower limb injuries and lower limb stress fractures during initial defence training^19^. Although promising, caution is required when interpreting these findings as the clinical trials included in the review were generally of low to moderate methodological quality. Nevertheless, a high quality randomised trial published after the systematic review mentioned above also found that prefabricated foot orthoses can reduce the rate of common lower limb overuse injuries by 34%^20^. The specific mechanism by which foot orthoses prevent injury is not fully established, although they have been shown to alter plantar pressure distribution, muscle activity and kinematics of the lower limb when used in conventional footwear^21^. However, at present, there is limited research on whether these same biomechanical effects also occur when foot orthoses are used in defence boots.

The primary aim of this study was to evaluate the effects of a contoured, prefabricated foot orthosis on plantar pressure and tibial acceleration while walking in defence boots. The secondary aim of this study was to evaluate the effects of the foot orthosis on footwear comfort. In doing so, our objective was to provide insights into the possible mechanisms by which contoured foot orthoses may reduce the risk of lower limb overuse injury in defence personnel.

## Methods

This study is reported in accordance with The Strengthening the Reporting of Observational Studies in Epidemiology (STROBE) statement^22^.

### Participants

The study was approved by the La Trobe University Human Ethics Committee (S15/202) and written informed consent was obtained from all participants. All experiments were performed in accordance with the World Medical Association’s Declaration of Helsinki. Twenty-eight adult participants from a local university were recruited between February and October 2017. Participants were excluded from the study if they had a history of foot surgery, an existing lower limb injury, or used any form of foot orthosis or shock-absorbing insole. Participant characteristics and anthropometric measures, including the Foot Posture Index (FPI-6)^23,24^ and navicular drop^25^, are displayed in Table 1.

**Table 1:** Characteristics of participants (*N* = 28).

Variable	Mean (SD)	Range
Age (years)	24.9 (5.3)	20.6 to 42.8
Sex, n (%) male	17 (60.7)	—
Height (cm)	175.0 (8.1)	162.0 to 190.0
Weight (kg)	75.6 (11.2)	50.0 to 100.0
Body mass index (kg/m^2^)	24.7 (2.9)	18.8 to 32.9
Waist circumference (cm)	83.7 (7.6)	67.0 to 100.0
Hip circumference (cm)	100.3 (4.8)	92.0 to 111.0
Waist to hip circumference ratio	0.83 (0.05)	0.73 to 0.94
Foot posture^†^	1.96 (1.9)	−1 to 6
Supinated, n (%)	1 (3.6)	—
Normal, n (%)	24 (85.7)	—
Pronated, n (%)	4 (14.3)	—
Navicular drop (mm)^25^	5.14 (3.7)	0 to 13
Ankle dorsiflexion, knee extended (degrees)^56^	44.6 (10.8)	31 to 63
Ankle dorsiflexion, knee flexed (degrees)^57^	46.4 (11.6)	32 to 64

^†^Foot posture was determined using the Foot Posture Index^24^, with scores between −12 (supinated characteristics) to +12 (pronated characteristics). Foot posture classified as supinated (FPI < 0), normal (FPI 0 to 5) or pronated (FPI > 5)^23^.

### Sample size

An *a priori* sample size calculation estimated that 28 participants were required to provide 80% power to detect a small effect size (0.25) for the three footwear-insole conditions, with statistical significance for hypothesis tests set at *p* < 0.05^26^.

### Contoured foot orthoses and flat insoles

A contoured, prefabricated foot orthosis and a flat insole were investigated in this study. The contoured foot orthosis and flat insole were manufactured by the same company (Foot Science International, Christchurch, New Zealand), were full-length and were made from the same material (firm single density, 140 kg/m^3^ closed cell polyethylene foam). The foot orthosis was contoured and varied in thickness, namely it was thicker in the region of the medial arch and around the periphery of the heel, whereas the flat insole had a uniform thickness of 3 mm^27^.

### Procedures

**Figure 1:**
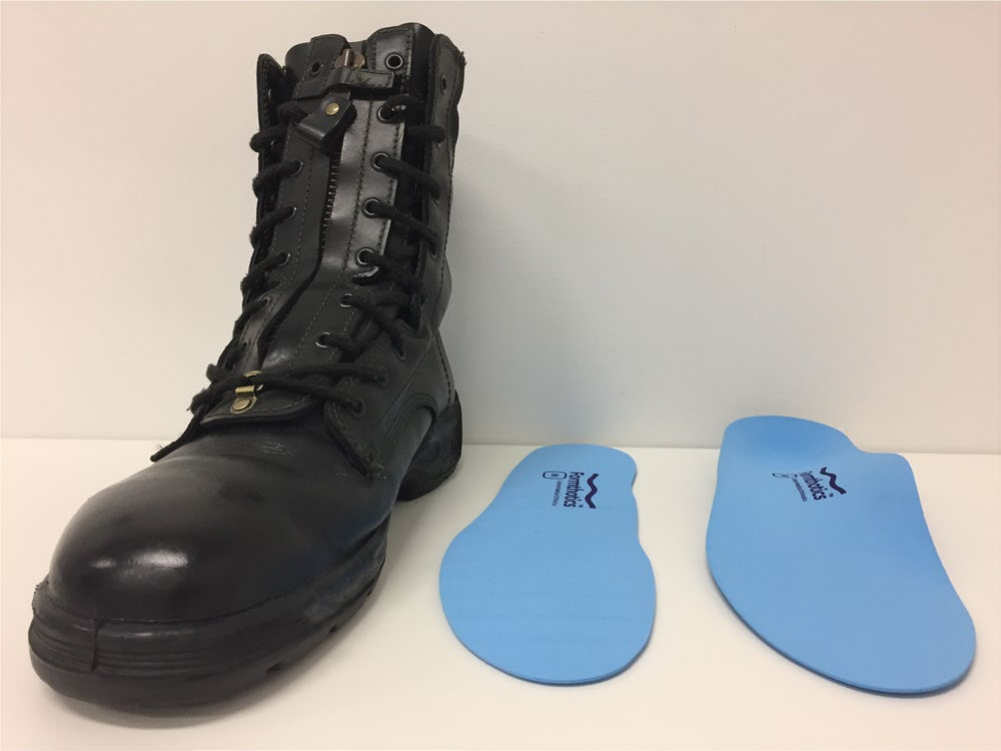
The three experimental conditions. Left to right: (i) Oliver defence boot; (ii) flat insole; (iii) contoured foot orthosis.

All data were collected at a single session at the same university from which the participants were recruited. Participants were advised that three footwear-insole conditions were being investigated during the study. The following three conditions (Fig. 1) were tested in random order (Research Randomizer, www.randomizer.org):

(i) Defence boot only (control)

(ii) Defence boot with flat insole

(iii) Defence boot with contoured foot orthosis

Each participant had a pair of the shoe insoles fitted to standard-issue Royal Australian Navy defence boots (Oliver Footwear® Style 66–395 230MM (9″) Lace up Structural Firefighter Boot, Ballarat, Australia). The shoe insoles were heat moulded to participants’ feet while wearing the boots. All participants wore standardised thin cotton socks (Bonds®, Kew, Melbourne, Australia) during testing. To maintain participant blinding, the boots were removed from the participants’ sight while the insoles were placed in them. The investigators were not blinded due to the difficulty in concealing the conditions, however this was not considered a limitation as the plantar pressure and accelerometer equipment provides objective data.

**Figure 2:**
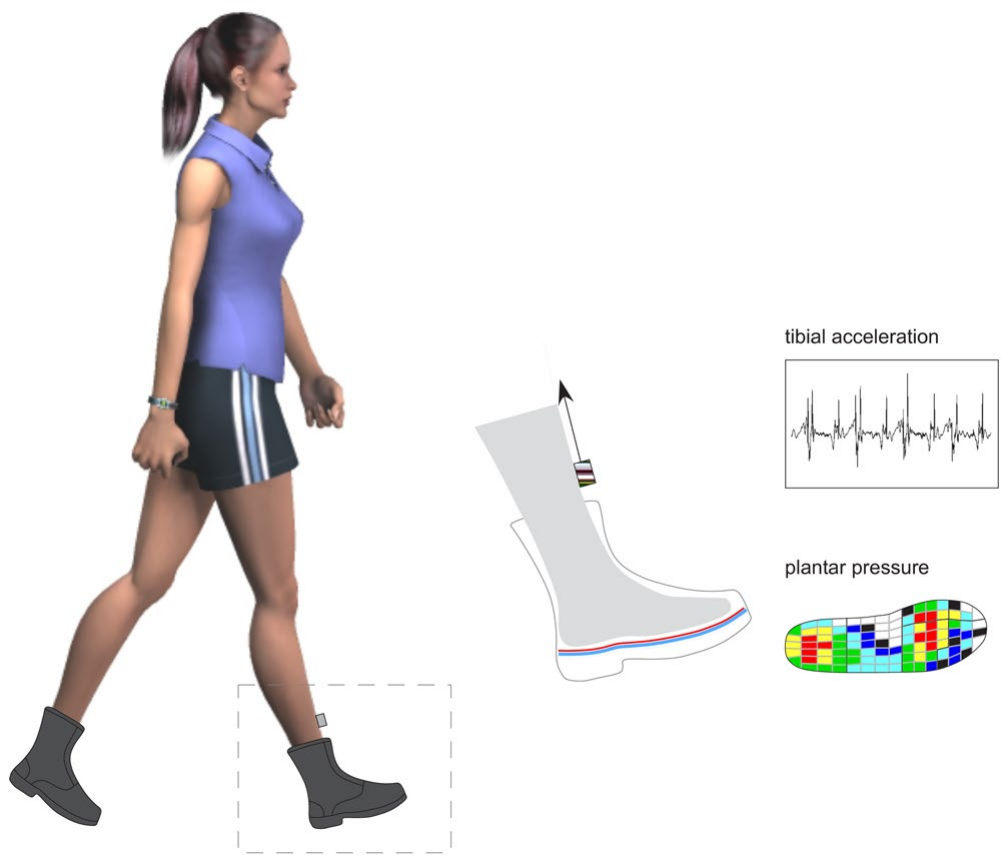
Plantar pressure and tibial acceleration data collection procedure.

Pressures beneath the foot were measured with the pedar^®^-X in-shoe system, which has been shown to be a valid, reliable and accurate measure of plantar pressure^28,29^. The pedar^®^-X insoles are thin, flexible and comprise 99 capacitive sensors. The pedar^®^-X insoles were calibrated using the trublu^®^ calibration device prior to data collection. Data were recorded at a frequency of 100 Hz. The pedar^®^-X insoles were placed within the boot, between the foot and the shoe insoles. Tibial accelerations were measured with a lightweight tri-axial accelerometer (measurement range: ± 16 g) (Biometrics Ltd, Newport, United Kingdom). The accelerometer was firmly secured to the anteromedial aspect of the participants’ right distal tibia, which provides an accurate measure of tibial acceleration^30,31^, and which has been shown to be highly correlated to vertical ground reaction forces experienced during gait^32,33^. Tibial accelerations were sampled at 1080 Hz (see Fig. 2).

After a familiarisation period of approximately 5 minutes in each condition, participants were timed as they completed 4 overground walking trials along an 8-metre walkway. Plantar pressure and tibial acceleration data were collected as participants walked at a comfortable self-determined speed. All trials were within 5% of the recorded walking time of the first trial^34^.

Following data collection for each condition, and while still blinded, participants rated the shoe comfort on a 100 mm visual analogue scale, with the left end (0 mm) labelled ‘not comfortable at all’ and the right end (100 mm) labelled ‘most comfortable imaginable’.

### Data analysis

The primary variables were: (i) plantar pressure (peak pressure, maximum force and contact area) under the rearfoot, midfoot and forefoot, and (ii) tibial peak positive acceleration. The secondary variables were foot contact time and footwear comfort.

For the plantar pressure and tibial acceleration data, only the middle 4 steps from each trial were included in the analysis. For each condition, a total of 16 steps (i.e. 4 steps from 4 trials) per participant were included in the analysis. To meet the independence assumption of statistical analysis, only data from the right limb were used^35^.

Plantar pressure data were analysed using the Novel-win program (version 20.3.30). Novel percent masks were applied to each individual footprint^36,37^. The rearfoot mask consisted of the proximal 30% of the foot length, the midfoot mask consisted of the middle 30% of the foot length and the forefoot mask consisted of the distal 40% of the foot length. The midfoot was further divided into lateral and medial halves. The forefoot mask consisted of the medial forefoot (1^st^ metatarsophalangeal joint region), lateral forefoot (including the 2^nd^ through 5^th^ metatarsophalangeal joint regions) and hallux. Acceleration data were initially filtered using a recursive low-pass Butterworth filter^38^. The acceleration data were then analysed using Python open-source software (Python Software Foundation, https://www.python.org/). An inbuilt function in the software was used to detect the tibial peak positive accelerations, defined as the highest positive acceleration. To achieve this, for each participant, the filtered and non-filtered data were visually inspected and the optimum cut off frequency was determined. This ranged between 100 to 200 Hz across all participants. The minimum distance between peaks was set at 800 frames for all participants.

Statistical analysis was performed using IBM SPSS Statistics 25 (IBM Corporation, Armonk, NY) computer program. The distribution of data were checked for normality. Some variables were positively skewed and therefore required logarithmic transformation prior to inferential analysis. A repeated measures analysis of variance (ANOVA) with Least Significant Difference (LSD) post hoc tests was used for comparison of the means between test conditions. Statistical significance for hypothesis tests was set at the conventional level of *p* < 0.05. Effect sizes for all significant main effects between the three conditions were calculated using Partial Eta Squared (*η*_p_^2^), and were interpreted as small (0.00 to <0.06), medium (0.06 to <0.14), and large (>0.14)^39^. Effect sizes were also reported for all significant pairwise comparisons using Cohen’s *d*^40^, and were interpreted as negligible (0 to <0.15), small (0.15 to <0.40), medium (0.40 to <0.75), large (0.75 to <1.10), and very large (>1.10).

## Results

**Figure 3:**
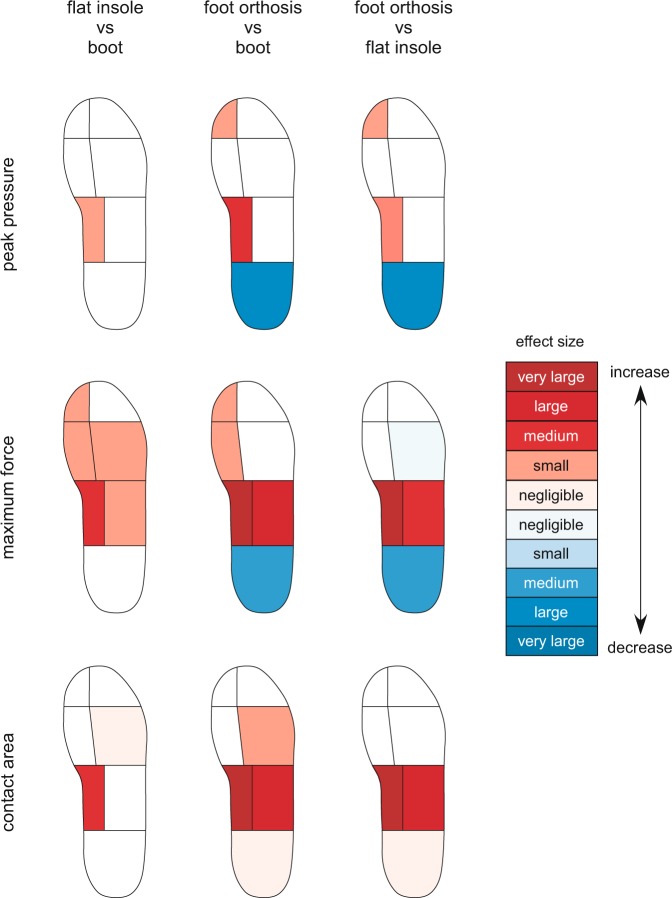
Effect size of changes in peak pressure, maximum force and contact area for each experimental condition compared to each other (*N* = 28).

Twenty-eight adult participants (17 men and 11 women) were recruited. The mean walking speed across all trials was 4.49 (±0.56) km/h (range 3.10 to 5.66 km/h). There were no significant differences in total foot contact time between the boot only condition (727.2 ± 61.6 ms), the flat insole (733.2 ± 70.9 ms, *p* = 0.208) and the foot orthosis (731.2 ± 64.0 ms, *p* = 0.233). There were a number of significant differences observed in plantar pressures (see Table 2 and Fig. 3) and tibial acceleration (see Table 3) between the three footwear-insole conditions being investigated.

**Table 2:** Comparisons of peak pressure, maximum force and contact area for each condition (*N* = 28).

Condition	Mean (SD)	Peak pressure (kPa)				Maximum force (N)			Mean (SD)	Contact area (cm^2^)		
		% change	Effect size (*d*)	*P*-value	Mean (SD)	% change	Effect size (*d*)	*P*-value		% change	Effect size (*d*)	*P*-value
***Rearfoot***												
Boot only (control)	214.1 (40.0)	n/a	n/a	n/a	545.3 (117.7)	n/a	n/a	n/a	43.5 (6.2)	n/a	n/a	n/a
Flat insole	213.9 (38.1)	0	−0.01	0.953	554.9 (126.6)	+2	0.08	0.193	43.5 (6.1)	0	0.00	0.971
Foot orthosis	172.5 (37.9)	−19	−1.07	<0.001*^#^	497.9 (116.6)	−9	−0.40	< 0.001*^#^	43.9 (6.2)	+ 1	0.06	0.010*^#^
***Medial midfoot***												
Boot only (control)	93.3 (42.4)	n/a	n/a	n/a	14.2 (10.1)^†^	n/a	n/a	n/a	4.0 (2.3)	n/a	n/a	n/a
Flat insole	108.4 (49.1)	+16	0.33	0.016*	19.6 (11.3)^†^	+38	0.50	<0.001*	5.3 (3.1)	+33	0.48	0.002*
Foot orthosis	128.1 (57.1)	+37	0.69	<0.001*^#^	47.4 (15.9)^†^	+334	2.49	<0.001*^#^	14.7 (3.4)	+368	3.69	<0.001*^#^
***Lateral midfoot***												
Boot only (control)	115.8 (29.0)^†^	n/a	n/a	n/a	115.1 (31.5)	n/a	n/a	n/a	28.3 (3.4)	n/a	n/a	n/a
Flat insole	115.9 (31.6)^†^	0	0.00	0.865	125.5 (44.2)	+9	0.27	0.014*	27.7 (5.7)	−2	−0.13	0.330
Foot orthosis	113.9 (32.1)^†^	−2	−0.06	0.508	143.9 (43.2)	+25	0.76	<0.001*^#^	31.6 (4.4)	+12	0.84	<0.001*^#^
***Medial forefoot***												
Boot only (control)	253.9 (58.5)	n/a	n/a	n/a	218.3 (65.2)	n/a	n/a	n/a	17.2 (2.6)	n/a	n/a	n/a
Flat insole	271.5 (73.2)	+7	0.27	0.033	237.6 (74.1)	+9	0.28	<0.001*	17.6 (2.6)	+2	0.15	0.058
Foot orthosis	279.6 (88.8)	+10	0.34	0.042	230.7 (72.9)	+6	0.18	0.017*	17.6 (2.6)	+2	0.15	0.038
***Lateral forefoot***												
Boot only (control)	249.5 (62.8)^†^	n/a	n/a	n/a	371.5 (99.5)	n/a	n/a	n/a	33.9 (4.9)	n/a	n/a	n/a
Flat insole	241.3 (53.4)^†^	−3	−0.14	0.127	392.2 (101.0)	+6	0.21	0.001*	34.6 (4.9)	+2	0.14	0.012*
Foot orthosis	238.8 (63.1)^†^	−4	−0.17	0.047	377.6 (109.6)	+2	0.06	0.449^#^	34.4 (4.8)	+1	0.10	0.038*
***Hallux***												
Boot only (control)	295.2 (117.4)	n/a	n/a	n/a	113.6 (49.3)	n/a	n/a	n/a	7.4 (1.3)	n/a	n/a	n/a
Flat insole	298.4 (90.4)	+1	0.03	0.774	123.9 (46.8)	+9	0.21	0.004*	7.6 (1.6)	+3	0.14	0.438
Foot orthosis	322.4 (105.8)	+9	0.24	0.026*^#^	121.8 (48.4)	+7	0.17	0.034*	7.4 (1.5)	0	0.00	0.888

Note: % change is relative to the boot only-control condition. ^†^Data transformed prior to determining significance. *Mean differences significant at the 0.05 level compared to the boot only condition. ^#^Mean differences significant at the 0.05 level compared to the flat insole condition.

**Table 3:** Peak positive tibial acceleration (g) for each condition (*N* = 28).

Condition	Mean (SD)	% change	Effect size (*d*)	*P*-value
Boot only (control)	2.22 (0.82)^†^	n/a	n/a	n/a
Flat insole	2.25 (0.89)^†^	+1.4	0.04	0.882
Foot orthosis	2.07 (0.75)^†^	−6.8	−0.19	0.018*^#^

Note: % change is relative to the boot only-control condition. ^†^Data transformed prior to determining significance. *Mean difference significant at the 0.05 level compared to the boot only condition. ^#^Mean difference significant at the 0.05 level compared to the flat insole condition.

### Plantar pressure

#### Rearfoot

There was a significant effect for peak pressure (Wilk’s Lambda = 0.13, *F* (2, 26) = 86.10, *p* < 0.001; effect size large, *η*_p_^2^ = 0.87, 90% confidence interval [CI] 0.76 to 0.90), maximum force (Wilk’s Lambda = 0.40, *F* (2, 26) = 19.58, *p* < 0.001; effect size large, *η*_p_^2^ = 0.60, 90% CI 0.35 to 0.70) and contact area (Wilk’s Lambda = 0.75, *F* (2, 26) = 4.29, *p* = 0.025; effect size large, *η*_p_^2^ = 0.25, 90% CI 0.02 to 0.42) between the three conditions. Compared to the boot only condition, the contoured foot orthosis provided significant effects on peak pressure (−19%, *p* < 0.001, effect size large; *d* = −1.07), maximum force (−9.0%, *p* < 0.001, effect size medium; *d* = −0.40) and contact area (+1%, *p* = 0.010, effect size negligible; *d* = 0.06), while the flat insole provided no significant effect on these variables. Compared to the flat insole, the contoured foot orthosis provided significant effects on peak pressure (−19%, *p* < 0.001, effect size large; *d* = −1.09), maximum force (−10%, *p* < 0.001, effect size medium; *d* = −0.47) and contact area (+1%, *p* = 0.018, effect size negligible; *d* = 0.06).

#### Medial midfoot

There was a significant effect for peak pressure (Wilk’s Lambda = 0.55, *F* (2, 26) = 10.53, *p* < 0.001; effect size large, *η*_p_^2^ = 0.45, 90% CI 0.17 to 0.59), maximum force (Wilk’s Lambda = 0.13, *F* (2, 26) = 85.55, *p* < 0.001; effect size large, *η*_p_^2^ = 0.87, 90% CI 0.76 to 0.90) and contact area (Wilk’s Lambda = 0.07, *F* (2, 26) = 169.87, *p* = 0.001; effect size large, *η*_p_^2^ = 0.93, 90% CI 0.87 to 0.95) between the three conditions. Compared to the boot only condition, the contoured foot orthosis provided significant increases on peak pressure (+37%, *p* < 0.001, effect size medium; *d* = 0.69), maximum force (+334%, *p* < 0.001, effect size very large; *d* = 2.49) and contact area (+368%, *p* < 0.001, effect size very large; *d* = 3.69). The flat insole also provided significant increases on peak pressure (+16%, *p* = 0.016, effect size small; *d* = 0.33), maximum force (+38%, *p* < 0.001, effect size medium; *d* = 0.50) and contact area (+33%, *p* = 0.002, effect size medium; *d* = 0.48). Compared to the flat insole, the contoured foot orthosis provided significant increases on peak pressure (+18%, *p* < 0.001, effect size small; *d* = 0.37), maximum force (+242%, *p* < 0.001, effect size very large; *d* = 2.02) and contact area (+277%, *p* < 0.001, effect size very large; *d* = 2.89).

#### Lateral midfoot

There was a significant effect for maximum force (Wilk’s Lambda = 0.31, *F* (2, 26) = 28.77, *p* < 0.001; effect size large, *η*_p_^2^ = 0.69, 90% CI 0.47 to 0.77) and contact area (Wilk’s Lambda = 0.46, *F* (2, 26) = 15.04, *p* = 0.001; effect size large, *η*_p_^2^ = 0.54, 90% CI 0.27 to 0.66) between the three conditions. Compared to the boot only condition, the contoured foot orthosis provided significant increases on maximum force (+25%, *p* < 0.001, effect size large; *d* = 0.76) and contact area (+12%, *p* < 0.001, effect size large; *d* = 0.84). The flat insole provided a significant increase on maximum force (+9%, *p* = 0.014, effect size small; *d* = 0.27), while having no significant effect on contact area (*p* = 0.330). Compared to the flat insole, the contoured foot orthosis provided significant increases on maximum force (+15%, *p* < 0.001, effect size medium; *d* = 0.42) and contact area (+14%, *p* < 0.001, effect size large; *d* = 0.77).

#### Medial forefoot

There was a significant effect for maximum force (Wilk’s Lambda = 0.61, *F* (2, 26) = 8.43, *p* = 0.002; effect size large, *η*_p_^2^ = 0.39, 90% CI 0.12 to 0.54) between the three conditions. Compared to the boot only condition, the contoured foot orthosis (+6%, *p* = 0.017, effect size small; *d* = 0.18) and flat insole provided a significant increase on maximum force (+9%, *p < *0.001, effect size small; *d* = 0.28).

#### Lateral forefoot

There was a significant effect for maximum force (Wilk’s Lambda = 0.56, *F* (2, 26) = 10.41, *p* < 0.001; effect size large, *η*_p_^2^ = 0.44, 90% CI 0.17 to 0.58) and contact area (Wilk’s Lambda = 0.79, *F* (2, 26) = 3.48, *p* = 0.046; effect size large, *η*_p_^2^ = 0.21, 90% CI 0.02 to 0.38) between the three conditions. Compared to the boot only condition, the contoured foot orthosis provided a significant increase on contact area (+1%, *p* = 0.038, effect size small; *d* = 0.10), while having no significant effect on maximum force (*p* = 0.449). The flat insole provided significant increases on maximum force (+6%, *p* = 0.001, effect size small; *d* = 0.21) and contact area (+2%, *p* = 0.012, effect size negligible; *d* = 0.14). Compared to the flat insole, the contoured foot orthosis provided a significant decrease on maximum force (−4%, *p* = 0.011, effect size negligible; *d* = −0.14).

#### Hallux

There was a significant effect for peak pressure (Wilk’s Lambda = 0.72, *F* (2, 26) = 5.19, *p* = 0.013; effect size large, *η*_p_^2^ = 0.29, 90% CI 0.04 to 0.45) and maximum force (Wilk’s Lambda = 0.73, *F* (2, 26) = 4.88, *p* = 0.016; effect size large, *η*_p_^2^ = 0.27, 90% CI 0.03 to 0.44) between the three conditions. Compared to the boot only condition, the contoured foot orthosis provided significant increases on peak pressure (+9%, *p* = 0.026, effect size small; *d* = 0.24) and maximum force (+7%, *p* = 0.034, effect size small; *d* = 0.17). The flat insole provided a significant increase on maximum force (+9%, *p* = 0.004, effect size small; *d* = 0.21), while having no significant effect on peak pressure (*p* = 0.774). Compared to the flat insole, the contoured foot orthosis provided a significant increase on peak pressure (+8%, *p* < 0.005, effect size small; *d* = 0.24).

### Tibial acceleration

There was a significant effect for tibial peak positive acceleration (Wilk’s Lambda = 0.70, *F* (2, 26) = 5.67, *p* = 0.009; effect size large, *η*_p_^2^ = 0.30, 90% CI 0.05 to 0.47) between the three conditions. Compared to the boot only condition, the contoured foot orthosis provided a significant decrease on tibial peak positive acceleration (−7%, *p* < 0.018, effect size small; *d* = −0.19), while the flat insole had no significant effect (*p* = 0.882). Compared to the flat insole, the contoured foot orthosis significantly decreased peak positive tibial acceleration (−8%, *p* < 0.016, effect size small; *d* = −0.22).

### Footwear comfort

There were no significant differences in footwear comfort (*p* = 0.842) between the boot only condition (63.2 ± 17.8 mm), the flat insole (65.8 ± 18.9 mm) and the contoured foot orthosis (64.0 ± 20.3 mm).

## Discussion

The aim of this study was to investigate the immediate effects of a contoured, prefabricated foot orthosis and a flat insole on plantar pressure, tibial acceleration and comfort while walking in defence boots. Our findings indicate that, compared to the defence boot only condition, the contoured foot orthosis provided a medium to large reduction on plantar pressure under the rearfoot, medium to very large increases in contact area and plantar pressures under the medial midfoot, and small increases in force under the medial forefoot and hallux. The flat insole also provided increases in contact area and plantar pressure under the medial midfoot, and small increases in force under the lateral forefoot, medial forefoot and hallux. Of note, the increases in contact area and plantar pressures observed with the flat insole under the medial midfoot were significantly less than those provided by the foot orthosis. Compared to the defence boot only condition, the contoured foot orthosis also provided a small reduction on tibial peak positive acceleration while no change was observed with the flat insole. Despite the different plantar pressure and tibial acceleration profiles, there were no differences in comfort on the day of testing between the three footwear-insole conditions.

The contoured shape of the foot orthosis resulted in a very large increase in contact area under the medial midfoot, which was approximately three-fold greater than the boot only condition and flat insole. This enabled the contoured foot orthosis to redistribute force over a greater area of the foot, thus providing large reductions on plantar pressures under the rearfoot. This finding is consistent with previous studies that have found that contoured foot orthoses reduce plantar pressure under the rearfoot^41,42^. However, another study found that the material from which the orthosis is manufactured, as well as its contour, influence plantar pressures at the midfoot and rearfoot^37^. Although the flat insole and the contoured foot orthosis used in our current study were made from the same material (140 kg/m^3^ closed cell polyethylene), the latter was substantially thicker in the region of the medial midfoot and around the periphery of the heel. When considering our findings in the context of the previous research^37,42^, it is likely that the plantar pressure changes observed in our study can be attributed to both the contour of the foot orthosis, and its thicker material under the midfoot and around the periphery of the heel.

The plantar pressure findings of this study, particularly the medium to large reductions in plantar loading under the rearfoot, may assist in explaining how contoured foot orthoses are effective in the prevention and treatment of some common lower limb overuse injuries^19,20,43^. For example, a prospective study found that Royal Marine recruits that had greater peak pressure under the rearfoot during barefoot running were at greater risk of developing a tibial stress fracture during initial defence training^44^. Further, a cross-sectional study found that recreational runners who developed plantar heel pain had greater rearfoot plantar loading compared to uninjured runners^45^. Regarding the treatment of injuries, a clinical trial has shown that a foot orthosis identical to the device used in this study was effective in the management of plantar heel pain^46^, while a more recent trial also found this orthosis reduced the incidence of plantar heel pain by 50% in naval recruits undertaking initial defence training^20^. It must also be noted that our data demonstrates small increases in force under the medial forefoot. This finding suggests caution is required when prescribing foot orthoses in defence boots as the increase in force under the medial forefoot may be of clinical importance and lead to the development of new pathologies in the forefoot, or exacerbate pre-existing pathologies. The greatest concern regarding increases in force under the forefoot is whether it increases the risk of stress fractures of the metatarsals, which have been shown to be a common site for such injuries among military personnel^47,48^. However, these fractures most commonly affect metatarsals 2–5, where the foot orthosis we tested provided no effect on plantar pressures, so it is unclear whether the changes we observed in forefoot loading are clinically meaningful.

The reduction in tibial peak positive acceleration provided by the contoured foot orthoses is also of interest, as tibial stress injuries, such as medial tibial stress syndrome and tibial stress fractures, are among the most common overuse injuries experienced by physically active defence personnel^1^. The tibias of individuals with tibial stress injuries have been shown to have less cortical bone (cross sectional area and thickness), which has been proposed to reduce the tibia’s ability to resist tibial loading and bending moments^49,50^. While bony structure is likely to contribute to the risk of developing tibial stress injury, the magnitude of loading applied to the tibia has also been identified as important. Runners with a history of tibial stress fractures have been shown to experience greater tibial accelerations compared to runners without injury^51–53^. The reduction in tibial acceleration observed in our study may assist in explaining the findings of a recent systematic review that found that foot orthoses reduced the risk of developing shin pain and tibial stress fractures in defence personnel by 73% and 35%^19^, respectively. It must be noted, however, that tibial stress injuries are most typically seen in individuals undertaking running-based activities, so it is possible that the effects that foot orthoses have on tibial acceleration are most relevant during running. Nevertheless, as tibial stress injuries are common in defence personnel, the immediate, albeit small, reduction in tibial peak positive acceleration when using contoured foot orthoses during walking in defence boots may be beneficial in the prevention of these injuries.

In addition to altering tissue loading, it has also been proposed that contoured foot orthoses can provide clinical benefits via improvements in comfort^54^. Of interest, our study found that the contoured foot orthosis, flat insole and defence boots were all perceived as being equally comfortable after a short period of use. From a practical standpoint, this would suggest that contoured foot orthoses may be used for the prevention and treatment of injury without having a detrimental effect on immediate comfort, which is likely to be an important consideration by defence personnel. It must be noted, however, that our study only measured immediate comfort and it is likely that comfort over an extended period of time, such as during prolonged standing or when completing a long march, is of greater importance to defence personnel.

The findings of this study need to be viewed in light of several key limitations. First, the pedar^®^-X can only record resultant force acting perpendicular to the pressure mapping insole. As the pedar^®^-X is unable to determine the shear component of forces, it is possible that some inherent measurement error occurs. Despite such limitations, the pedar^®^-X provides a valid, reliable and accurate measure of plantar pressure and it is considered the best available method for measuring forces acting between the foot and shoe insoles^28,29^. Second, while the shoe insoles did provide some effect on plantar pressure and tibial acceleration, extrapolating these findings to kinematic changes is inherently speculative, although it has been proposed that the biomechanical effects of foot orthoses result from changes in plantar loading^54,55^. Third, it remains unknown what effects the shoe insoles have on plantar pressure, tibial acceleration and comfort over the longer term as a result of material compression and acclimatisation. Fourth, although participants were blinded during testing we did not measure how successful this was. As such, some of the differences observed in our study may have resulted, in part, from non-specific effects. Fifth, the participants in this study were healthy (uninjured) and relatively young, and although this is representative of military recruits, it remains unclear if the effects observed in this study may differ among military personnel, who may also be older and / or injured. Sixth, as the participants in our study were only observed during comfortable walking it would be beneficial for future studies to investigate the effects of foot orthoses on biomechanical outcomes during other tasks commonly performed by military personnel, such as marching, during load-carriage, and when in a fatigued state. Finally, we recognise that the association between changes to plantar pressures, tibial accelerations and injury has not been fully established in a military setting. In consideration of the aforementioned limitations, it would be beneficial for future studies to investigate if the changes in biomechanical variables, including plantar pressure and tibial acceleration, have an effect on injury incidence in military personnel.

In summary, the findings of this study indicate that contoured, prefabricated foot orthoses provide significant increases in plantar pressures at the midfoot and force at the medial forefoot, and reductions in plantar pressures at the rearfoot, while also providing small reductions in tibial peak positive acceleration. The flat insole also increased medial midfoot loads, but to a much lesser extent compared to the foot orthosis, while having no effect on tibial peak positive accelerations. These findings provide useful insights into the mechanisms that may be responsible for foot orthoses reducing the rate of lower limb overuse injury in defence personnel.

## Data Availability

The data generated during and/or analysed during the current study are available from the corresponding author on reasonable request.
